# Personals

**Published:** 1913-02

**Authors:** 


### PERSONALS.

Dr. A. S. Couch of Fredonia was the guest of honor at the Masonic Club rooms in January. In commemoration of the 50th anniversary of his service as officiating master of Forest Lodge, F. & A. M., he was presented with congratulations engrossed on parchment and with a past master's jewel.

Dr. William M. Hamilton has been elected Mayor of St. Catherines.

---

Dr. Rene Gaultier, our Associate Editor, of Paris, has been spending some time in Rome, whence he sends greetings.

---

Dr. A. F. Luhr of Buffalo has moved to 22 N. Pearl St., the former location of Dr. Keiser. He will devote his attention to the eye, ear, nose and throat.

---

Dr. W. E. Dake announces the removal of his office to 174 East Avenue, Rochester.

---

Dr. Joseph C. O'Gorman of Buffalo has received the permanent appointment of Medical School Examiner at a salary of $1000.

---

Dr. Henry Smoyer, City Treasurer of North Tonawanda, is making an extended tour of the West Indies. He will visit Dr. H. C. Leonhardt of Porto Rico, formerly of North Tonawanda.

---

Dr. E. B. Becker of Summerville, Mass., visited his family in Buffalo during the holidays.

---

Dr. Pemberton Lundy has been appointed Health Commissioner of North Tonawanda.

---

Major Wm. G. Bissell, M. D., City Bacteriologist of Buffalo, has returned after taking a post graduate course at the Army Medical School at Washington.

---

Dr. W. J. Moore of Westfield has been spending some weeks at Atlantic City.

---

Dr. T. M. Johnson of Buffalo has been elected Surgeon of Bidwell-Wilkeson Post, G. A. R.

---

Dr. H. H. Glosser of Buffalo has recently returned from a trip to North Carolina.

---

Dr. Henry D. Wilson, formerly of Boston, has been assigned to the Naval Recruiting Station at Buffalo, in place of Dr. E. L. Bogart.

Dr. G. H. Sherman of Detroit, the manufacturer of vaccines, has been in London, Eng., for a month.

---

Dr. Roswell Park of Buffalo is out of town.

---

Dr. L. H. Snow of Jamestown was wounded in the face in a collision between his auto and a street car, January 3.

---

Dr. C. E. Rose of Buffalo had about $150 worth of jewelry stolen from his home on the night of December 23.

---

Dr. Lucien Howe of Buffalo delivered a popular lecture at the University Building, January 12, on the "Progress of Medicine and Surgery."

---

Dr. W. H. Annowski of Buffalo is about to take a trip to Florida.

---

Dr. Alice Parker of Chicago recently visited her son, Dr. R. Emerson Parker of Jamestown.

---

Dr. C. Everett Field of Brooklyn, though still in private practice, has recently left the advertising department of Kress & Owen, to take the presidency of the Holetheol Co., 24 Cliff St., N. Y. Dr. Field is a member of the A. M. A. and its component societies and is a founder and the President of the People's Forum, as well as the organizer of Temple Forum.

---

Dr. Richard Kovacs of 236 E. 69th St., N. Y. City, is organizing a Physicians' Travel-Study tour.

---

Dr. J. C. Clemesha of Buffalo is in southern California, expecting to return about February 10.

---

Rev. Philip L. Frick, D. D., of the Delaware Avenue M. E. Church, Buffalo, delivered an address on Dr. Grenfell, Christian Physician, January 19, in a course on "The Acts of Some Modern Apostles."

---

Dr. John L. Eckel announces the opening of his office at 143 Allen St., Buffalo. Practice limited to mental and nervous diseases.

---

Dr. Yamei Kin of China lectured in Buffalo on the social status of women in China, last month. She was the guest of Dr. and Mrs. W. H. Marcy.

Dr. A. T. Leonard of North Tonawanda is in Hot Springs, Ark.

Dr. Willes E. Ford of Utica has resigned as medical director of St. Luke's Hospital, but will remain a member of the board. Mr George W. Wilson, late Asst. Supt. of Roosevelt Hospital, will succeed him.

